# Dietary L-arginine supplementation reduces Methotrexate-induced intestinal mucosal injury in rat

**DOI:** 10.1186/1471-230X-12-41

**Published:** 2012-04-30

**Authors:** Tal Koppelmann, Yulia Pollak, Jorge Mogilner, Jacob Bejar, Arnold G Coran, Igor Sukhotnik

**Affiliations:** 1 The Bruce Rappaport Faculty of Medicine, Technion-Israel Institute of Technology, Laboratory of intestinal adaptation and recovery, Haifa, Israel; 2 Department of Pediatric Surgery, Bnai Zion Medical Center, Haifa, Israel; 3 Department of Surgery, Bnai Zion Medical Center, Haifa, Israel; 4 Department of Pathology, Bnai Zion Medical Center, Haifa, Israel; 5 Section of Pediatric Surgery C.S. Mott Children's Hospital and University of Michigan Medical School, Ann Arbor, MI, USA

**Keywords:** Methotrexate, Mucositis, Intestine, Arginine, Rat

## Abstract

**Background:**

Arginine (ARG) and nitric oxide maintain the mucosal integrity of the intestine in various intestinal disorders. In the present study, we evaluated the effects of oral ARG supplementation on intestinal structural changes, enterocyte proliferation and apoptosis following methotrexate (MTX)-induced intestinal damage in a rat.

**Methods:**

Male rats were divided into four experimental groups: Control rats, CONTR-ARG rats, were treated with oral ARG given in drinking water 72 hours before and 72 hours following vehicle injection, MTX rats were treated with a single dose of methotrexate, and MTX-ARG rats were treated with oral ARG following injection of MTX. Intestinal mucosal damage, mucosal structural changes, enterocyte proliferation and enterocyte apoptosis were determined 72 hours following MTX injection. RT-PCR was used to determine bax and bcl-2 mRNA expression.

**Results:**

MTX-ARG rats demonstrated greater jejunal and ileal bowel weight, greater ileal mucosal weight, greater ileal mucosal DNA and protein levels, greater villus height in jejunum and ileum and crypt depth in ileum, compared to MTX animals. A significant decrease in enterocyte apoptosis in the ileum of MTX-ARG rats (vs MTX) was accompanied by decreased bax mRNA and protein expression and increased bcl-2 protein levels.

**Conclusions:**

Treatment with oral ARG prevents mucosal injury and improves intestinal recovery following MTX- injury in the rat.

## Background

Mucositis is a debilitating, dose-limiting, and costly side effect of cancer therapy. Mucositis occurs in 40% of cancer patients after standard doses of treatment and in almost 100% of patients treated with high doses of chemotherapy [1] and can affect the entire gastrointestinal tract causing discomfort, nausea, vomiting, bloating, diarrhoea, ulceration, bleeding and in some cases result in septicaemia [2]. The mechanisms of mucositis are poorly understood. It has been suggested that proinflammatory cytokines, e.g., TNF-α and IL-1β, may be involved in the amplification phase of intestinal mucositis [3].

Arginine is a non-essential amino acid processed metabolically by the urea cycle and involved in multiple metabolic and biologic processes including release of several hormones, immune response, the regulation of inflammation, collagen synthesis during wound healing, and tumor biology [4]. L-Arginine is the precursor for the formation of nitric oxide (NO), an important signaling molecule involved in neurotransmission, vascular homeostasis, immune regulation, and host defense [5]. In addition, arginine is an essential metabolic substrate for immune cells and required for normal lymphocyte function [6]. Arginine and NO are critical to the normal physiology of the gastrointestinal tract. Several studies have suggested that endogenous formation of nitric oxide maintains the mucosal integrity of the intestine and protects the gut from injuries from blood-borne toxins and tissue destructive mediators [7,8]. In a recent study, we have shown that oral arginine decreases intestinal injury caused by lipopolysacharide endotoxemia in a rat [9]. In another study, we have demonstrated that oral arginine administration improves mucosal recovery following intestinal ischemia/reperfusion injury in the rat [10]. In a recent review, Drover et al. have described that perioperative administration of arginine supplemented diets in elective surgical patients results in a substantial reduction in infectious complications and shorter hospital length of stay, with no overall effect on mortality compared with standard care [11].

The purpose of this study was to evaluate the effectiveness of dietary L-arginine in prevention of methotrexate (MTX) induced intestinal damage in a rat model and to investigate the mechanisms involved in the stimulating effect of arginine on enterocyte turnover including its effects on cell proliferation and cell death via apoptosis.

## Methods

### Animals

The experimental protocol was approved by the "Guide for the Care and Use of Laboratory Animals," Rappaport Faculty of Medicine, Technion (Haifa, Israel). Male Sprague-Dawley rats weighing 250-300 g were acclimatized at 21°C on 12-h day and night cycles for a minimum of 1 week before experimentation. The rats had free access to water and were pair-fed with standard chow.

### Experimental design

Animals were randomly assigned to one of four experimental groups of 8 rats each: (1) sham rats (Group A), (2) sham-ARG rats were pretreated with ARG given in drinking water (2%) for 7 days (Group B), (3) MTX rats, which underwent methotrexate injury by a single intraperitoneal injection of 20 μg/kg and were sacrificed 3 days later (Group C), and (4) MTX-ARG animals, which were pretreated with ARG given in drinking water 3 days before and 3 days after MTX injection (Group D).

### Intestinal morphology analysis

The small intestine was removed and divided into two segments: proximal jejunum (10 from the Treitz ligament) and terminal ileum (10 cm from ileo-cecal junction). Each segment was weighed, mucosa was scraped off, weighed and snap frozen in liquid nitrogen. Mucosal samples (100 mg) were homogenized using Kontes Tenbroeck Tissue Grinder. DNA and protein were extracted using TRIZOL reagent as described by Chomczynski [12]. The DNA concentrations were recorded spectrophotometrically and calculated per cm of bowel length. Final protein concentration was measured spectrophotometrically using a commercially available kit (Bio-Rad, Protein Assay) and was calculated per cm of bowel length. Histological sections were prepared from the jejunal and ileal remnants. Pieces of proximal jejunum and terminal ileum near the ileo-cecal junction were placed in 5% phosphate-buffered formalin, washed with absolute alcohol, and embedded in paraffin, cut to 5 (m thickness, and stained with hematoxylin-eosin. Fifteen of longest villi and crypts were selected for the microscopic analysis, using a 10 × 4 magnifying lens.

### Enterocyte proliferation and apoptosis

Crypt cell proliferation was assessed using a biotinylated monoclonal anti-BrdU antibody system provided in a kit form (Zymed Laboratories, Inc, San Francisco, CA). An index of proliferation was determined as the ratio of crypt cells staining positively for BrdU per 10 crypts. Additional 5 μm thick sections were prepared to establish the degree of enterocyte apoptosis. Immunohistochemistry for Caspase-3 (Caspase-3 cleaved concentrated polyclonal antibody; dilution 1:100; Biocare Medical, Walnut Creek, CA) was performed for identification of apoptotic cells using a combination of streptovidin-biotin-peroxidase method and microwave antigen retrieval on formalin-fixed, paraffin-embedded tissues according to the manufacturers' protocols. For each group, the number of stained cells was counted in at least ten villi. The apoptotic index (AI) was defined as the number of apoptotic cells per ten villi. All measurements were performed by a qualified pathologist blinded as to the source of intestinal tissue.

### Expression of bax and bcl-2 genes (real-time PCR)

Expression of Bax and Bcl-2 levels was determined by quantitative real-time PCR (7500 Real-Time PCR System, Applied Biosystems, USA) on cDNA samples using Cyber Green Master Mix (ROVALAB, Germany) with the exception of template and primers. Primers for Rattus norvegicus Bax and Bcl-2 were synthesized by Syntezza Bioscience ltd. Israel, and 18 s rRNA Control kit from Eurogentec, EGT Group.

### Western blotting

Tissue from jejunum and ileum was homogenized in RIPA lysis buffer containing 50 mM Tris-HCl (pH 7.4), 150 mM NaCl, 1% NP-40, 2 mM EDTA, supplemented with a cocktail of protease and phosphatase inhibitors. Protein concentrations were determined by Bradford reagent according to the manufacturer's instructions. Samples containing equal amounts of total protein (30 μg) were resolved by SDS-PAGE under reducing conditions. After electrophoresis, proteins were transferred to a PVDF membrane and probed with various primary antibodies to anti-Bcl-2 antibody (1:1000 dilution, sc-7382), anti-Bax antibody (1:200 dilution, sc-493), anti-phospho-ERK antibody (1:2500 dilution, sc-7383), anti-β-catenin antibody (1:1000 dilution, sc-7199) and anti-ERK2 antibody (1:1000 dilution, sc-56899). Horseradish peroxidase-conjugated secondary antibody was purchased from Jackson ImmunoResearch Laboratories Inc. (West Grove, PA) and an enhanced chemiluminescent substrate from Biological Industries (Kibbutz Beth HaEmek, Israel). The optical density of the specific protein bands was quantified by using a densitometer (Vilber Lourmat, Lion, France).

### Statistical analysis

The data are expressed as the mean ± SEM. A one-way ANOVA for comparison, followed by Tukey's test for pair-wise comparison was used for statistical analysis. Prism software was used (GraphPad Software, Inc., San Diego, CA) and statistical significance was defined as *P *< 0.05.

## Results

### Water, arginine and energy intake

Total water intake was 15-18 ml/rat/day. There was no significant difference in fluid intake between all experimental groups (15 ml/rat/day in control animals and 18 ml/rat/day in MTX-groups). The amount of additional protein was 300 mg/rat/a day and additional calorie intake was 1.2 kcal/rat/day.

### Body weight changes

MTX (Group C) rats demonstrated a significant decrease in final body weight (100 ± 2.6 vs 106 ± 0.4%initial weight, p < 0.05) compared to control animals (Group A). Although treatment with oral arginine resulted in a trend toward increase in final body weight in both sham (Group B) and MTX (Group D) animals, this trend did not achieve statistical significance.

### Intestinal mucosal parameters

Oral arginine administration in control animals (Group B) resulted in a mild decrease in bowel weight and mucosal weight in jejunum and ileum, as well as mucosal DNA content (33%, p < 0.05) in jejunum compared to control animals (Table 1). MTX-induced intestinal damage (Group C) was associated with a significant decrease in bowel weight in jejunum (26%, p < 0.05) and ileum (20%, p < 0.05), mucosal weight in jejunum (two-fold decrease, p < 0.05) and ileum (two-fold decrease 25%, p < 0.05), mucosal DNA in jejunum and ileum (two-fold decrease, p < 0.05), and mucosal protein in jejunum (38%, p < 0.05) and ileum (41%, p < 0.05) compared to control animals (Group A). Following dietary L-arginine administration (Group D), MTX-rats demonstrated a significant increase in bowel weight in jejunum (12%, p < 0.05), bowel (24%, p < 0.05) and mucosal (30%, p < 0.05) weight in ileum, ileal mucosal DNA (two-fold increase, p < 0.05) and protein (59%, p < 0.05) compared to MTX-untreated animals (Group C).

**Table 1:** Intestinal mucosal parameters

	CONTR	CONTR-ARG	MTX	MTX-ARG
Bowel weight				

(mg/cm/100 mg body weight)				

Jejunum	23 ± 1	21 ± 1	17 ± 0.3 *	19 ± 1 *†

Ileum	21 ± 1	19 ± 1	17 ± 1 *	21 ± 1 *†

Mucosal weight				

(mg/cm/100 mg body weight)				

Jejunum	9.4 ± 0.5	7.6 ± 0.5 *	5.1 ± 0.6 *	6.2 ± 0.6

Ileum	9.1 ± 0.5	6.7 ± 0.5 *	4.7 ± 0.5 *	6.1 ± 0.6 *†

Mucosal DNA				

(μg/cm/100 mg body weight)				

Jejunum	45 ± 7	30 ± 4 *	22 ± 5 *	25 ± 3 *

Ileum	47 ± 6	52 ± 4	19 ± 4 *	38 ± 9 †

Mucosal protein				

(μg/cm/100 mg body weight)				

Jejunum	94 ± 21	60 ± 11	58 ± 10 *	58 ± 5 *

Ileum	54 ± 10	44 ± 8	32 ± 5 *	51 ± 8 †

Villus height (μm)				

Jejunum	434 ± 29	345 ± 56	314 ± 33 *	413 ± 18 †

Ileum	371 ± 26	287 ± 19 *	217 ± 52 *	300 ± 23 *†

Crypt depth (μm)				

Jejunum	183 ± 17	131 ± 8 *	112 ± 4 *	126 ± 11

Ileum	161 ± 16	120 ± 8 *	101 ± 15 *	131 ± 10 *†

CONTR- control, MTX-methotrexate, ARG-arginine

* P < 0.05 All groups vs CONTR rats, † P < 0.05 MTX-ARG vs MTX rats.

### Microscopic bowel appearance

**Figure 1:**
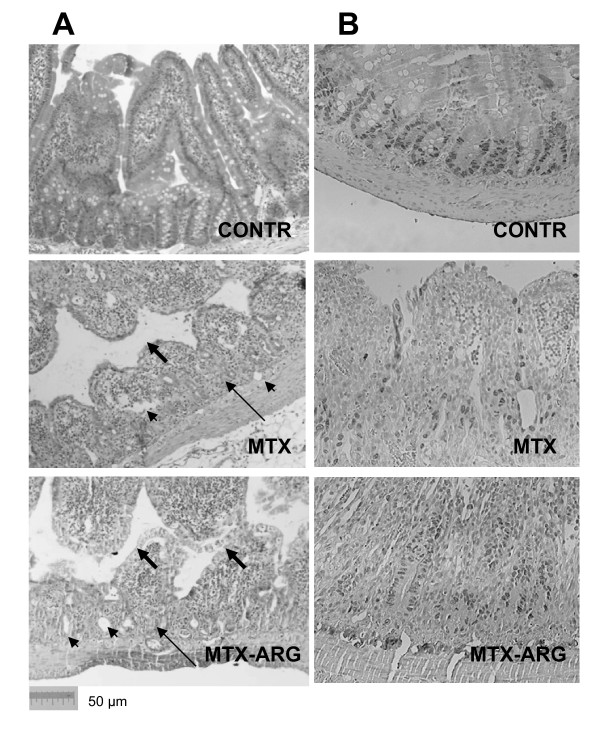
**A. Staining with H@E. Intestinal injury caused by MTX**. MTX rats demonstrated a significant epithelial atrophy (thick arrows) and signs of crypt remodeling (thin arrows) which were accompanied by marked cellularity and an increased number of blood vessels (arrowheads) in the stroma. Following ARG administration rats showed less significant epithelial atrophy and crypt remodeling compared to MTX rats. **B**. BrdU staining (cell proliferation). Control rats show normal crypt compartment. The proliferative zone in MTX-rats moved progressively upwards in the crypts toward the crypt-villus junction. The proliferative zone of MTX-ARG rat was only mildly affected showed the presence of newly formed crypts and signs of regeneration. CONTR-control, MTX-methotrexate, ARG-arginine.

MTX rats showed severe villous atrophy, epithelial flattening, extensive crypt loss and signs of crypt remodeling, which was accompanied by marked cellularity and an increased number of blood vessels in the stroma (Figure 1A). The proliferative zone in MTX-rats moved progressively upwards in the crypts toward the crypt-villus junction (Figure 1B). At the same time, the proliferative zone of MTX-ARG rat was only mildly affected, showing a slight shift upwards within the crypts. Although the mucosa was still severely damaged, MTX-ARG rats showed the presence of newly formed crypts and regeneration.

Villus height and crypt depth significantly decreased in jejunum (28% and 39%, correspondingly, p < 0.05) and ileum (42% and 37%, correspondingly, p < 0.05) in MTX-rats compared to control animals (Table 1). Dietary L-arginine exerted different effects on the microscopic intestinal appearance in control and MTX animals. In control rats, oral arginine (Group B) resulted in a mild but significant decrease in villus height and crypt depth in jejunum and ileum compared to control animals (Group A). In MTX rats, dietary L-arginine (Group D) attenuated the negative effect of methotrexate on mucosal architecture. MTX-ARG rats showed greater villus height in jejunum (32%, p < 0.05) and ileum (38% increase, p < 0.05), and crypt depth in ileum (30% increase, p < 0.05) compared to MTX-animals (Group C).

### Cellular proliferation and apoptosis

CONTR-ARG rats showed a trend toward a decrease in cell proliferation; however, this trend was not statistically significant (Figure 1). MTX intestinal damage (Group C) resulted in a significant decrease in enterocyte proliferation rates in jejunum (128 ± 12 vs 197 ± 14 BrdU positive cells/10crypts, p < 0.05) and ileum (133 ± 21 vs 210 ± 22 BrdU positive cells/10crypts, p < 0.05) when compared to control animals (Group A). Although treatment with dietary arginine of MTX-animals (Group D) resulted in a 10% increase in cell proliferation rates in jejunum and ileum, this change was not statistically significant.

The frequency of apoptotic cells was increased in the animals following MTX-induced damage in jejunum (1.8 ± 0.2 vs 0.8 ± 0.37 apoptotic cells/1- villi, p < 0.05) and ileum (1.5 ± 0.3 vs 0.6 ± 0.13 apoptotic cells/1- villi, p < 0.05) compared to control rats (Figure 1). Treatment with dietary arginine of MTX-animals (Group D) resulted in a significant two-fold decrease in cell apoptosis in both jejunum and ileum (p < 0.05) compared to MTX-nontreated animals (Group C).

### Expression of apoptosis related genes

**Figure 2:**
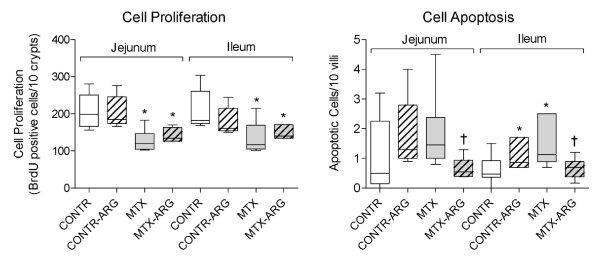
**Effects of MTX and arginine on crypt cell proliferation and apoptosis**. The number of labeled cells in 10 well-oriented, longitudinal crypts per section from each rat was determined using light microscopy. Identification of apoptotic cells was performed using immunohistochemistry for Caspase-3. The apoptotic index is expressed as the percentage of apoptotic cells per 10 villi. Values are mean ± SEM. CONTR- control, MTX-methotrexate, ARG-arginine. * P < 0.05 MTX and MTX-ARG vs CONTR rats, † P < 0.05 MTX-ARG vs MTX rats.

**Figure 3:**
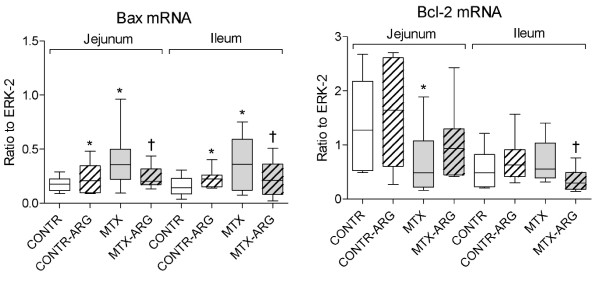
**Effect of arginine on bax and bcl-2 mRNA expression in gut mucosa following methotrexate induced intestinal damage**. Results are expressed as the ratio of the investigated bax and bcl-2 mRNA to ERK. Values are mean ± SEM. CONTR- control, MTX-methotrexate, ARG-arginine. * P < 0.05 MTX and MTX-ARG vs CONTR rats, † P < 0.05 MTX-ARG vs MTX rats.

CONTR-ARG (Group B) rats showed a small but significant increase in bax mRNA (pro-apoptotic factor) expression in jejunum (33%) and ileum (53%) compared to control animals (Figure 2). MTX rats (Group C) demonstrated a two-fold increase in bax mRNA expression in jejunum and ileum (p < 0.05) as well as a two-fold decrease in bcl-2 gene (anti-apoptotic protein) expression (p < 0.05) in jejunum compared to control animals (Group A). Treatment with arginine resulted in a significant decrease in bax mRNA expression in jejunum (38%, p < 0.05) and ileum (41%, p < 0.05) and a concomitant decrease in bcl-2 gene expression in ileum (44%, p < 0.05) compared to MTX animals (Figure 3).

### Western blotting

**Figure 4:**
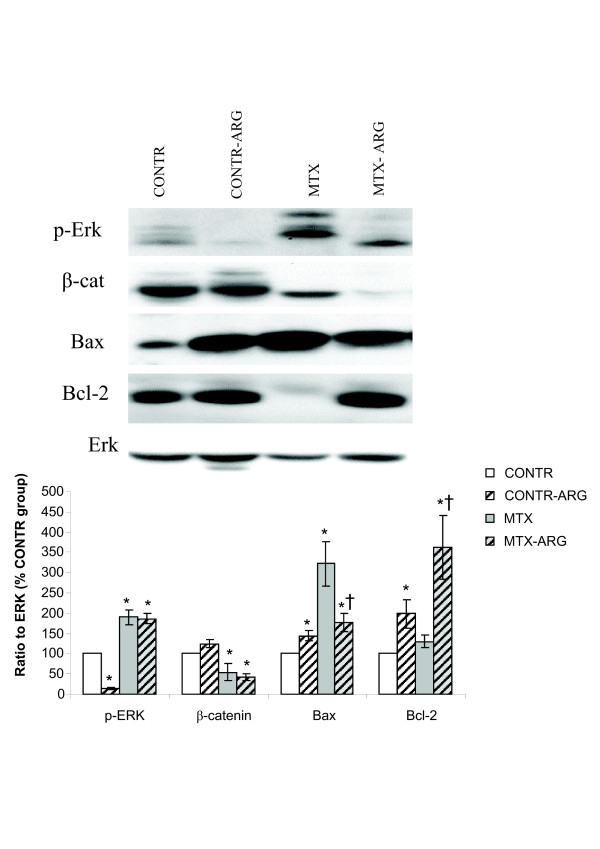
**Effect of MTX and dietary arginine on β-catenin, p-ERK, bax and bcl-2 protein levels in ileum (Western blotting)**. Values are presented as a ratio to ERK protein level and are expressed as a percentage of the control group. CONTR- control, MTX-methotrexate, ARG-arginine.

Although western blot analysis of proliferation and apoptosis markers has demonstrated a similar tendency both in jejunum and ileum, more representative changes were observed in the terminal ileum. Western blot analysis (Figure 4) illustrated a significant decrease in bcl-2 protein levels as well as a significant increase in bax protein in MTX rats compared to control animals. These findings correlate with augmented cell apoptosis in MTX-animals compared to control rats. Decreased cell proliferation in this group coincided with decreased β-catenin levels. Treatment with arginine resulted in a significant increase in bcl-2 protein levels as well as a significant decrease in bax protein levels without changes in p-ERK and β-catenin levels compared to MTX rats. These qualitative and quantitative changes are in agreement with the mRNA expression data and with decreased apoptosis and unchanged proliferation in ileum following ARG administration.

## Discussion

In the last two decades, arginine has attracted major interest since it has been identified as the natural substrate of nitric oxide, and is now recognized to play a major role in many regulation processes. Nitric oxide is a multifunctional intercellular messenger molecule that plays an important role in a variety of physiological processes. Nitric oxide is synthesized from L-arginine by the inducible nitric oxide synthase (iNOS). Arginine and nitric oxide are critical to the normal physiology of the gastrointestinal tract and maintain the mucosal integrity of the intestine in various intestinal disorders. Arginine stimulates intestinal cell migration and intestinal protein synthesis through a focal adhesion kinase dependent mechanism in vitro [13]. Arginine supplementation enhances intestinal growth and development in weanling piglets [14]. Dietary supplementation with arginine stimulates small intestinal mucosal recovery following experimental radiation enteritis [15] and accelerates ulcer healing in experimental ulcerative ileitis [16]. In a recent study, we have shown that oral arginine decreases intestinal injury caused by lipopolysacharide endotoxemia in a rat [9]. In another study, we have demonstrated that oral arginine administration improves mucosal recovery following intestinal ischemia-reperfusion injury in a rat [10]. The mechanisms of these positive effects are still unclear; however, a stimulating effect of appropriate amounts of NO on enterocyte proliferation and a suppressive effect on enterocyte death via apoptosis may be considered as one of them.

The role of arginine in prevention of chemotherapy-induced intestinal damage is unclear. Hämäläinen Met al have shown recently that chemotherapy inhibites iNOS expression, and subsequent NO production, in a dose-dependent manner at therapeutically achievable drug concentrations in a human colon epithelial cell line [17]. In a recent experiment, Gulgun et al. have demonstrated that proanthocyanidin, arginine and glutamine supplementation had a positive effect in the protection of the small intestine from methotrexate-induced injury [18]. The mechanisms of this effect were not investigated. In a recent clinical trial, Izaola et al. have shown that arginine and glutamine enhanced formula decreases the rate of radiotherapy-induced oral mucositis in patients with head and neck cancer [19].

Methotrexate is the most commonly used anti-metabolite agent in clinical oncology practice. Since ARG maintains the mucosal integrity of the intestine in various intestinal disorders, we hypothesized in the present study that this amino acid could prevent intestinal mucosal injury or/and improve intestinal recovery following MTX-induced mucositis. Alterations in bowel and mucosal weights, mucosal DNA and protein content and histological appearance were measured. BrdU was used in our experiment to determine an index of crypt cell proliferation. Immunohistochemistry for caspase-3 was used to characterize enterocyte apoptosis. We have demonstrated that a single dose of MTX caused a severe mucosal injury in the small bowel, indicated by severe villous atrophy, epithelial flattening, intensive crypt loss and signs of crypt remodeling, which was accompanied by marked cellularity and an increased number of blood vessels in stroma. In addition, treatment with MTX resulted in significant mucosal hypoplasia. A decrease in bowel and mucosal weight, mucosal DNA and protein, and decrease in villus height support this conclusion. Parallel decreases in mucosal DNA and protein indicate that the smaller mucosal mass of MTX animals can be attributed to cellular hypoplasia. Histologically, villus height decreased in response to MTX administration, suggesting decreased absorptive surface area. Mucosal DNA content as well as the enterocyte proliferation index decreased significantly in both jejunum and ileum following MTX administration. MTX is an analog of folic acid and works by attaching to dehydrofolate reductase to inhibit DNA production. As a result crypt proliferation in the small bowel is inhibited. In addition, the proliferative zone in MTX-rats moved progressively upwards in the crypts toward the crypt-villus junction. The mechanism responsible for this effect is poorly understood. Verburg and coworkers have shown in MTX-treated rats that BrdU-positive cells are not restricted to the crypts but are also found in up to one-third of the length of the villi due to migration of the cells [20]. Our data suggest that MTX animals had lower β-catenin levels which fits with decreased cell proliferation. Extensive experimental evidence suggests that Wnt/β-catenin signaling plays a central role in maintaining the intestinal stem-cell niche and in regulating differentiation of stem cells within the intestinal epithelium toward either enterocytes or one of three secretory cell lineages (Paneth, goblet, or enteroendocrine cells [21]. Cell loss in the small intestine MTX-induced mucositis is mainly regulated by programmed cell death. Our results show that the intrinsic pathway, with its regulation by the bcl-2 family of proteins, was altered by MTX consistent with changes in cell apoptosis: the mRNA levels of the pro-apoptotic gene bax increased, while those of the antiapoptotic bcl-2 gene decreased. With regard to the protein level, qualitative and quantitative changes were in agreement with mRNA expression. Bax protein levels were significantly up-regulated while bcl-2 protein levels were down regulated in MTX-treated rats compared to control animals. These changes correlate with the enhanced enterocyte apoptosis during MTX-induced mucositis.

Results of the present study show that dietary arginine protects the intestinal mucosa from damage caused by MTX. While MTX rats showed severe villous atrophy, epithelial flattening, extensive crypt loss and signs of crypt remodeling, marked cellularity and an increased number of blood vessels in stroma, arginine-treated rats showed more preserved architecture as well as the presence of newly formed crypts and regeneration. While the proliferative zone in MTX-rats moved progressively upwards in the crypts toward the crypt-villus junction, the proliferative zone of MTX-ARG rats was only mildly affected, showing a slight shift upwards within the crypts. In addition, exposure to oral arginine significantly enhanced intestinal recovery following MTX-induced damage. This is evident from the significant increase in bowel and mucosal, increased DNA and protein content in ileum. Increased mucosal weight without changes in mucosal surface area may suggest that mucosal hyperplasia rather than bowel enlargement or intestinal muscle hypertrophy is responsible for the increased intestinal mass. Increase in mucosal DNA and protein along with hypertrophy of the individual cells which we have demonstrated morphometrically is characteristic of tissues undergoing increased cell proliferation or repair. Histologically, marked increases in villus height in both jejunum and ileum suggest increased absorptive surface area and closely correlate with increased cell mass. The present data suggest that arginine did not change significantly mucosal proliferation in functioning intestine, but decreased significantly cell apoptosis rate, which may represent the main mechanism that maintains mucosal structure following MTX-induced damage. Our results show that the intrinsic pathway, with its regulation by the bcl-2 family of proteins, was altered by arginine in accordance with changes in cell apoptosis: the mRNA and protein levels of the pro-apoptotic bax decreased, while those of the antiapoptotic bcl-2 protein levels increased. Correspondingly, bax/bcl-2 ratio decreased in MTX-ARG rats compared to MTX animals, suggesting increased enterocyte survival. Further investigation is needed to define the regulation of this special apoptotic state with respect to the Fas/Fasl-mediated extrinsic pathway.

The mechanism of the positive effects of arginine is unclear. In non-operated, non-stressed animal, total food intake is 20-25 g/rat/day, total protein intake is about 6 g/rat/day, and total energy intake is 100 kcal/rat/day [22]. We do not believe that an additional 300 mg/rat/day of protein (vs 6 g of total protein) as well as additional energy of 1.2 kcal/rat (vs total 100 kcal/rat/day) may have influenced the results. The limitation of the current experiment is a lack of isonitrogenous MTX group. Therefore, this study is not able to prove that arginine supply limits intestinal damage. It was previously reported that other amino acids (like glutamine, glycine or histidine) have protective effects on intestinal mucosa during chemotherapy or inflammatory conditions. An additional MTX-isonitrogenous amino acid group will be added in future study to determine whether other amino acids have similar effects.

## Conclusions

In a rat model of methotrexate-induced mucositis, administration of dietary arginine prevents intestinal damage, decreases cell death via apoptosis and accelerates intestinal recovery. These preliminary observations suggest that oral arginine may have clinical value in preventing or reducing the severity of chemotherapy-induced mucositis.

## Competing interests

The authors declare that they have no competing interests.

## Authors' contributions

TK was responsible for conception and participation in design, experimental work and collection of data, analysis and interpretation of results, drifting and substantial editing the manuscript. YP was responsible for experimental work and collection of data, analysis and interpretation of results. JM was responsible conception and participation in design, analysis and interpretation of results. JB was responsible for experimental work and collection of data, analysis and interpretation of results. AGC participated actively in the conceptualization of the paper, analyses plan and provided critical reviews on the manuscripts. IS participated conceptualization, interpretation of the findings, drafting and editing of the manuscript. All authors read and approved the final manuscript.

## Pre-publication history

The pre-publication history for this paper can be accessed here:

http://www.biomedcentral.com/1471-230X/12/41/prepub
